# Endometrial ablation as a safe treatment for adenomyosis-related abnormal uterine bleeding in women with cerebral infarction: a case report

**DOI:** 10.3389/fmed.2025.1708029

**Published:** 2025-11-05

**Authors:** Limei Zheng, Hong Zhang, Wei Fang, Jianhua Yang

**Affiliations:** ^1^Department of Obstetrics and Gynecology in Sir Run Run Shaw Hospital, School of Medicine, Zhejiang University, Key Laboratory of Reproductive Dysfunction Management of Zhejiang Province, Hangzhou, China; ^2^Department of Obstetrics and Gynaecology, People's Hospital of Putuo District, Zhoushan, China; ^3^Department of Neurology, Hangzhou Hospital of Traditional Chinese Medicine, Affiliated to Zhejiang Chinese Medical University, Hangzhou, Zhejiang, China

**Keywords:** adenomyosis, cerebral infarction, abnormal uterine bleeding, endometrial ablation, hysteroscopy

## Abstract

**Background:**

Adenomyosis is often associated with abnormal uterine bleeding (AUB), which can lead to anemia. Recurrent bleeding, especially in the presence of cerebral infarction risk, may exacerbate the likelihood of cerebral infarction through a hypercoagulable state. Although the relationship between adenomyosis and ischemic stroke is less frequently explored, controlling AUB and maintaining antithrombotic therapy remain clinical challenges. This report presents the application of endometrial ablation in a patient with adenomyosis, AUB, and cerebral infarction.

**Methods:**

We report a case of a 50-year-old female patient who developed acute ischemic stroke due to adenomyosis-related AUB. During the acute phase, the patient underwent endometrial ablation, successfully controlling the bleeding while continuing antiplatelet therapy.

**Results:**

After endometrial ablation, the patient's abnormal uterine bleeding was effectively controlled, with no recurrence of cerebral infarction. At a 37-month follow-up, the patient had no further vaginal bleeding or recurrence of cerebral infarction.

**Conclusion:**

This case is the first to report the use of endometrial ablation in a patient with adenomyosis and cerebral infarction, demonstrating the potential of this procedure in emergency hemostasis and maintaining antithrombotic therapy. Endometrial ablation is an effective, minimally invasive treatment with a quick recovery, making it a viable option for patients who cannot tolerate major surgery.

## 1 Introduction

Adenomyosis is a common benign gynecological disorder characterized by the presence of endometrial glands and stroma within the myometrium. Typical symptoms include AUB, menorrhagia, dysmenorrhea, and secondary anemia, which significantly impair patients' quality of life. Current treatment strategies include pharmacological therapy, minimally invasive procedures, and surgical interventions, but treatment options remain challenging, particularly for patients with severe comorbidities.

Recent studies have suggested a potential link between adenomyosis and ischemic stroke. The underlying mechanisms include a hypercoagulable state, chronic inflammation, and blood flow changes associated with anemia. However, this relationship has not been extensively studied, and clinical reports remain limited. In particular, managing abnormal bleeding while maintaining antithrombotic therapy poses a major clinical challenge for patients with cerebral infarction. Traditional treatments such as GnRH agonists or hysterectomy are often limited due to their relatively low efficacy, side effects, or the risks associated with surgery.

In contrast to existing treatment options, which often involve hysterectomy or conservative management, this case report presents the first use of endometrial ablation as a uterine-preserving strategy. Endometrial ablation is a minimally invasive procedure that effectively reduces uterine bleeding, with a rapid recovery and low complication rate. However, no reports have described its use in patients with adenomyosis and cerebral infarction.

In this case report, we present the first case of a patient with adenomyosis complicated by AUB and cerebral infarction, who successfully underwent emergency endometrial ablation for hemostasis and experienced long-term clinical benefits. This study aims to evaluate the feasibility and potential clinical value of this approach in complex cases and to provide new insights into the management of similar patients.

## 2 Case report

A 50-year-old woman presented with involuntary tremors, transient weakness in the left lower limb, and facial asymmetry. She also reported a history of heavy, irregular vaginal bleeding over the past month.

**Family history:** Her father had hypertension and died from cerebral hemorrhage; her mother had hypertension and ischemic stroke; and her brother has hypertension.

**Physical examination:** Respiratory rate: 19 breaths/min; temperature: 37.2°C; pulse: 106 bpm; blood pressure: 138/85 mmHg. The patient was alert, mildly lethargic, oriented, and had normal strength (grade 5) in all limbs with no obvious neurological deficits (NIHSS score 0).

**Figure 1:**
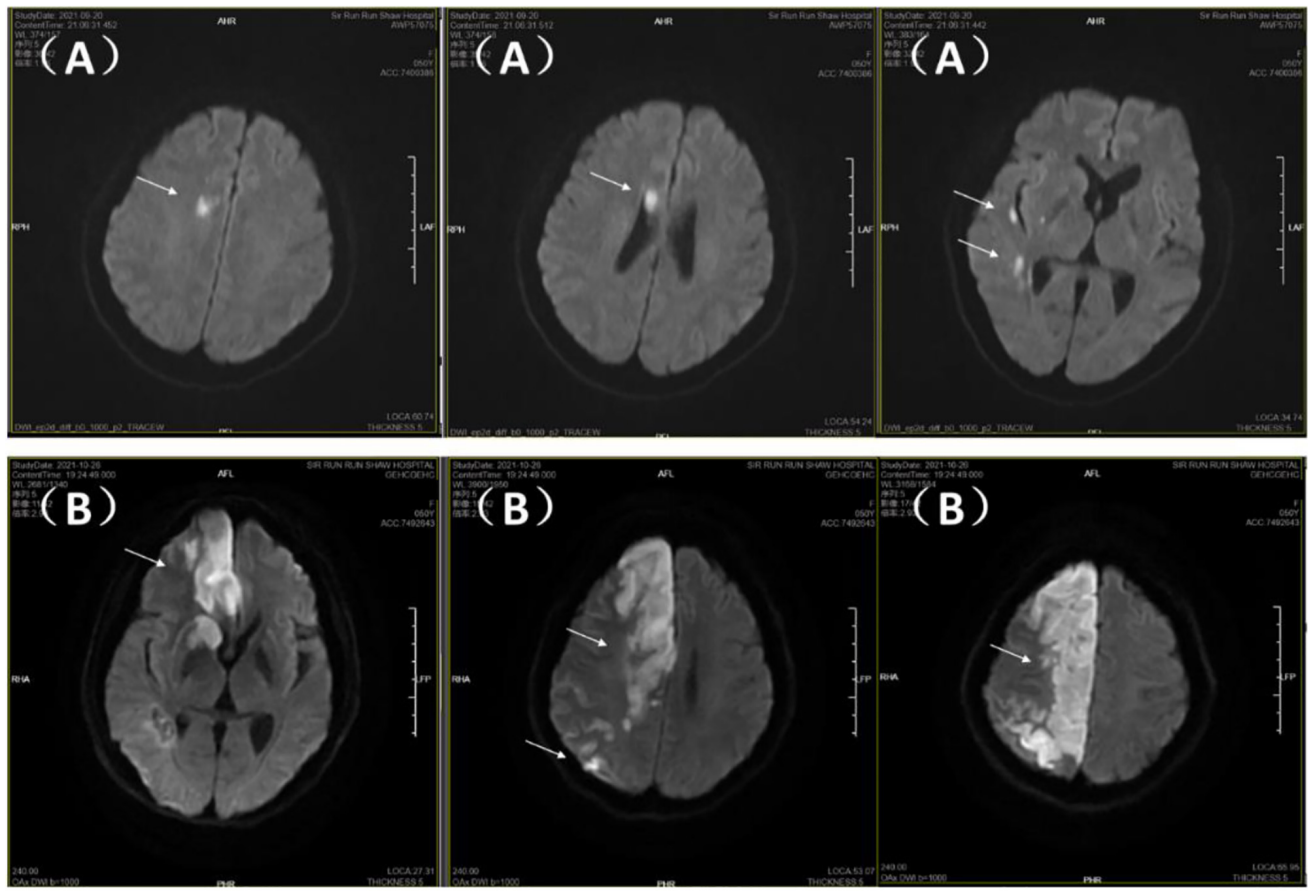
Brain MRI-DWI sequences. **(A)** Initial admission: multiple small infarcts in right frontotemporal region, basal ganglia, and periventricular area (arrows). **(B)** Readmission: progression to extensive right hemispheric paramedian infarction (arrows).

**Laboratory and imaging studies:** Hemoglobin was 86 g/L. Brain MRI with DWI showed multiple recent infarcts in the right hemisphere (Figure 1A). Cranial CTA revealed moderate stenosis in the left middle cerebral artery (M1 segment) and mild stenosis in the left posterior cerebral artery (P2 segment). Brain and chest CT scans were normal. Serum CA125 was 26.8 U/mL; D-dimer was 0.23 μg/mL. The lipid profile, antinuclear antibodies, vasculitis panel, erythrocyte sedimentation rate (ESR), protein S, and protein C were all negative. Lower limb venous ultrasound showed no thrombosis. Echocardiogram and 24-h Holter monitoring revealed no abnormalities. Transvaginal ultrasound indicated adenomyosis with an enlarged uterus (9.00 × 7.68 × 8.74 cm), thickened endometrium (1.17 cm), and heterogeneous echogenicity predominantly in the posterior myometrium (maximal thickness 4.91 cm).

**Initial diagnosis:** Right-sided ischemic stroke; AUB; adenomyosis; moderate anemia.

**Clinical course:** The patient was treated with dual antiplatelet therapy (aspirin and clopidogrel), oxytocin, and transfusion. On day 4, persistent vaginal bleeding and worsening limb weakness were noted, and hemoglobin dropped to 66 g/L. Clopidogrel was discontinued, and dilation and curettage (D&C) was performed. Histopathology confirmed simple endometrial hyperplasia, excluding carcinoma and atypical hyperplasia. Post-procedure, blood transfusion and cessation of dual antiplatelet therapy led to improved bleeding control, and hemoglobin rose to 75 g/L. Limb strength also improved. Aspirin was restarted on postoperative day 2, with further recovery of muscle strength (right limbs grade 5, left upper limb grade 4+, left lower limb grade 4). The patient was discharged on day 18, ambulating independently.

**Figure 2:**
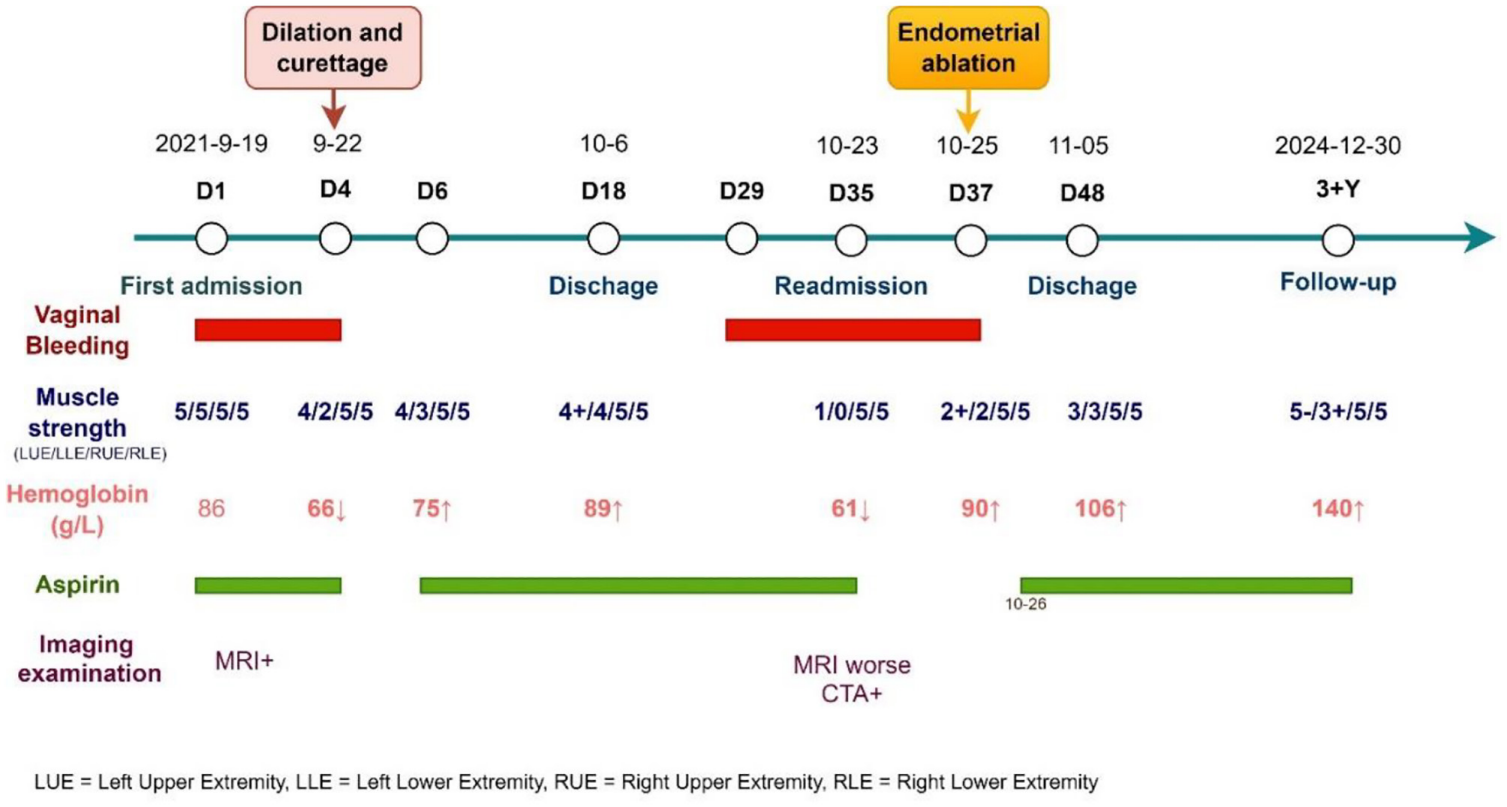
Clinical timeline of the index patient.

**Readmission:** 17 days later, the patient was readmitted with heavy vaginal bleeding. Neurological examination revealed grade 1 strength on the left side and mixed aphasia. CTA showed stenosis and distal occlusion of the right anterior cerebral artery (A2 segment) and severe distal stenosis in the right middle cerebral artery. The patient declined thrombolysis or thrombectomy due to surgical risks. Repeat DWI showed a large, progressing infarct in the right hemisphere near the midline (Figure 1B). Emergency hysteroscopic endometrial ablation was performed, successfully stopping the bleeding. Antiplatelet therapy (aspirin) was resumed postoperatively. No further vaginal bleeding occurred, anemia improved (hemoglobin 14 g/L), and there was no recurrence of ischemic stroke or vaginal bleeding at 37 months of follow-up (see Figure 2 for the treatment algorithm). The patient was satisfied with the treatment.

## 3 Discussion

This case report highlights the unique application of endometrial ablation in a patient with adenomyosis complicated by cerebral infarction. Adenomyosis is frequently associated with AUB and anemia, and it has also been linked to a hypercoagulable state (1), which may increase the risk of ischemic stroke. Although the association between adenomyosis and cerebral infarction has not been extensively investigated, existing literature has suggested several mechanisms, including mucin-related hypercoagulability (2), persistent inflammation of ectopic endometrium (3, 4), as well as anemia and acute hemorrhagic states (5). Together, these factors may promote thrombus formation and increase the likelihood of cerebrovascular events. Anemic patients often exhibit elevated erythropoietin (EPO) levels, leading to secondary thrombocytosis. Alterations in red blood cell deformability may impair oxygen delivery and disrupt blood flow, further exacerbating cerebral hypoxia. Additionally, anemia-induced hypoxia can cause endothelial dysfunction, resulting in ischemic brain tissue damage (5). In our patient, CA125 and D-dimer levels were within normal ranges; however, the onset of ischemic stroke occurred in the context of acute bleeding and intracranial arterial stenosis. The temporal correlation between disease progression and vaginal bleeding suggests that hemorrhage may have served as a trigger for cerebral infarction. This finding indicates that the hypercoagulable state in such patients may be underestimated. Therefore, even in the presence of normal CA125 and D-dimer levels, clinicians should remain vigilant for stroke risk in patients with abnormal bleeding, anemia, and vascular stenosis.

The patient initially presented to the neurology department, where, despite the absence of limb symptoms, MRI confirmed cerebral infarction. Dual antiplatelet therapy was initiated early to prevent progression (6), without consultation from a gynecologist. However, the patient's vaginal bleeding worsened, hemoglobin continued to decline, and conservative treatments such as transfusion and fluid replacement were ineffective. The patient's worsening neurological symptoms raised concerns about anemia-related hypoperfusion. Consequently, antiplatelet therapy was temporarily discontinued, and gynecological intervention was sought to control the bleeding. Following dilation and curettage, bleeding improved and aspirin was restarted for secondary stroke prevention. During this period, vaginal bleeding was closely monitored, and the patient's condition gradually improved. Subsequently, the patient was readmitted due to acute vaginal bleeding and stroke. Given the patient's history and considering vaginal bleeding as a trigger for recurrence, aspirin was stopped to reduce bleeding risk, and after endometrial ablation, vaginal bleeding completely subsided. Antiplatelet therapy was then resumed. Patients with this type of presentation often first consult neurology and may lack experience with gynecological management. This case underscores the importance of multidisciplinary collaboration, as early intervention may improve patient outcomes.

In the acute phase, the patient faced the dual challenge of abnormal uterine bleeding and the risk of cerebral infarction. The most common treatment options currently available include GnRH agonists therapy (7) and total hysterectomy (8). However, GnRH agonists may worsen the condition due to flare effects in the short term, and recurrence is common after discontinuation (8–12). Although hysterectomy is effective, it is highly invasive. In the acute phase, prolonged discontinuation of antithrombotic therapy may place the patient at risk for recurrent stroke or other thrombotic events, while performing surgery during this phase can lead to complications such as intra-abdominal hemorrhage (13) and pulmonary embolism (14). Therefore, endometrial ablation, as a minimally invasive procedure with quick recovery and minimal trauma (15), offered a feasible alternative for this patient. Previous studies have reported successful use of endometrial ablation in patients with vaginal bleeding complicated by coagulopathy (16, 17), showing good hemostatic outcomes. Therefore, we also attempted this procedure in our patient. The procedure successfully achieved hemostasis while allowing continued antithrombotic therapy, thus preventing further cerebrovascular events. In contrast, hysterectomy is not a viable option for patients wishing to preserve fertility. The following table compares the clinical characteristics and outcomes of our case with similar cases from existing literature, highlighting the differences and similarities in the treatment approaches and outcomes observed in patients with adenomyosis-associated ischemic stroke (Table 1). Additionally, high-quality educational videos may help alleviate patients' fears and misconceptions about the procedure (18).

**Table 1:** Clinical characteristics and outcomes of patients with adenomyosis-associated ischemic stroke.

**Case**	**Author, year**	**Age**	**Country**	**Primary department**	**Clinical presentation**	**Menstrual period**	**CA-125 (U/mL)**	**D-dimer (μg/mL)**	**Hb (g/L)**	**NBTE**	**Extracerebral infarction**	**Treatment for cerebral infarction**	**Treatment for adenomyosis**	**Hysterectomy**	**Recurrence**	**Reference number**
1	Okazaki K, 2018	42	Japan	Neurology	Hemiparesis, aphasia	No	395	1.4	–	No	No	Warfarin	–	No	No	(2)
2	Okazaki K, 2018	50	Japan	Neurology	Hemiparesis, aphasia	No	143	3.7	–	No	No	Rivaroxaban	–	No	No	(2)
3	Yin X, 2018	46	China	Neurology	Hemiplegia	Yes	546.5	12	121	No	No	–	–	Yes	No	(11)
4	Aiura R, 2021	48	Japan	Neurology	Fever, impaired consciousness	Yes	3,536.2	79.3	82	No	Bilateral kidneys	UFH → Edoxaban, endovascular thrombectomy	–	Yes	No	(14)
5	Arai N, 2022	50	Japan	Neurology	Hemianopsia	Yes	999	6.4	92	No	No	UFH → Apixaban	GnRH agonist	Yes	Yes → No	(8)
6	Yasuda M, 2022	47	Japan	Neurology	Hand weakness, aphasia	Yes	90.3	3.8	113	No	Kidney	UFH → Edoxaban	–	Yes	Yes → No	(13)
7	Morishima Y, 2023	42	Japan	Neurology	Ataxia	Yes	576	9.7	132	No	Bilateral kidneys	UFH → Edoxaban	GnRH agonist	Yes	Yes → No	(12)
8	Present case, 2024	50	China	Neurology	Ataxia in left upper limb	No	26.8	0.23	8.6	No	No	Antiplatelet	D&C → Endometrial ablation	No	Yes → No	-

Source references correspond to the main text citations (2, 8, 11–14).

CA-125, cancer antigen 125; D&C, dilation and curettage; GnRH, gonadotropin-releasing hormone; Hb, hemoglobin; NBTE, nonbacterial thrombotic endocarditis; UFH, unfractionated heparin.

Notes: Normal reference ranges: CA-125 <35 U/mL; D-dimer <0.5 μg/mL; Hemoglobin 12–16 g/dL (female); “Yes → No” indicates initial recurrence followed by resolution after definitive treatment; “–” indicates data not available in the original report.

In this case, endometrial ablation not only effectively controlled abnormal bleeding but also ensured uninterrupted antiplatelet therapy, which may have contributed to reducing the risk of recurrent infarction. During a 37-month follow-up period, the patient experienced neither recurrent stroke nor vaginal bleeding, demonstrating favorable long-term outcomes. This suggests that endometrial ablation may serve as an effective therapeutic option for high-risk patients who cannot tolerate more invasive procedures, particularly during the acute phase, by balancing hemostatic control with the need for ongoing antithrombotic therapy. Endometrial ablation effectively reduces menstrual bleeding but results in significant endometrial destruction, leading to loss of fertility potential. Unlike hysterectomy, it preserves the uterus anatomically but is not suitable for patients who wish to conceive (19).

Although this is the first report describing the application of endometrial ablation in a patient with adenomyosis and cerebral infarction, and the subsequent follow-up indicated a favorable prognosis, the generalizability of this approach remains limited. Further studies with larger patient cohorts are required to evaluate the safety, efficacy, and long-term outcomes of endometrial ablation in similar patient populations, and to determine its potential role as a standard therapeutic option.

The limitations of this study include the fact that it is based on a single case and lacks large sample data. Additionally, adenomyosis was diagnosed using imaging, without histological confirmation. As the disease is rare, although there have been multiple reports, there is still a lack of basic research and confirmatory studies to clearly establish the causal relationship between adenomyosis and ischemic stroke.

## 4 Conclusion

This case report presents the first exploration of endometrial ablation in patients with adenomyosis complicated by cerebral infarction. The procedure effectively controlled abnormal uterine bleeding while maintaining antithrombotic therapy. Thirty-seven months post-surgery, the patient showed no recurrence of cerebral infarction or abnormal uterine bleeding, suggesting that the procedure offers favorable long-term outcomes. It provides an effective approach for achieving rapid hemostasis and preserving antithrombotic treatment, with minimal trauma and quick recovery. While the evidence is currently limited, the procedure holds potential clinical value for high-risk patients and warrants further investigation.

## Data Availability

The datasets generated and/or analyzed during the current study are not publicly available due to privacy and confidentiality restrictions but are available from the corresponding author on reasonable request.
